# Comparison of Overall Survival between Surgical Resection and Radiofrequency Ablation for Hepatitis B-Related Hepatocellular Carcinoma

**DOI:** 10.3390/cancers13236009

**Published:** 2021-11-29

**Authors:** Moon Haeng Hur, Jeong-Hoon Lee, Ju Yeon Kim, Ji Hoon Hong, Min Kyung Park, Hee Jin Cho, Na Ryung Choi, Jihye Kim, Minseok Albert Kim, Joon Yeul Nam, Yun Bin Lee, Eun Ju Cho, Su Jong Yu, Yoon Jun Kim, Dong Ho Lee, Jeong Min Lee, Suk Kyun Hong, Nam-Joon Yi, Kwang-Woong Lee, Kyung-Suk Suh, Jung-Hwan Yoon

**Affiliations:** 1 Department of Internal Medicine and Liver Research Institute, Seoul National University Hospital, Seoul National University College of Medicine, Seoul 03080, Korea; mhhur@snu.ac.kr (M.H.H.); kimjuyean@snu.ac.kr (J.Y.K.); toyfriend7@snu.ac.kr (J.H.H.); alsrud627@snu.ac.kr (M.K.P.); 74792@snuh.org (H.J.C.); 74599@snuh.org (N.R.C.); 74788@snuh.org (J.K.); linojade@snu.ac.kr (M.A.K.); moreno1@snu.ac.kr (J.Y.N.); yblee@snu.ac.kr (Y.B.L.); creatio3@snu.ac.kr (E.J.C.); ydoctor2@snu.ac.kr (S.J.Y.); yoonjun@snu.ac.kr (Y.J.K.); yoonjh@snu.ac.kr (J.-H.Y.); 2 Department of Radiology, Seoul National University Hospital, Seoul National University College of Medicine, Seoul 03080, Korea; dhlee.rad@snu.ac.kr (D.H.L.); jmsh@snu.ac.kr (J.M.L.); 3 Department of Surgery, Seoul National University Hospital, Seoul National University College of Medicine, Seoul 03080, Korea; hsk0831@snu.ac.kr (S.K.H.); gsleenj@snu.ac.kr (N.-J.Y.); kwleegs@snu.ac.kr (K.-W.L.); kssuh@snu.ac.kr (K.-S.S.)

**Keywords:** liver cancer, curative treatment, hepatitis B virus, nucleos(t)ide analogue, entecavir, tenofovir

## Abstract

**Simple Summary:**

The effectiveness of surgical resection and radiofrequency ablation in early hepatocellular carcinoma is still controversial because previous studies show conflicting results. In addition, previous studies did not consider the antiviral treatment-related factors, even though there is now robust evidence that antiviral therapy is crucial for determining the prognosis of patients with chronic hepatitis B-related liver cancer. After adjusting for the antiviral treatment, we demonstrated that radiofrequency ablation may provide comparable overall survival to resection in the treatment of very early or early hepatocellular carcinoma, although recurrence-free survival is marginally shorter than in the resection group.

**Abstract:**

It remains controversial whether surgical resection, compared to radiofrequency ablation (RFA), improves overall survival (OS) in patients with early hepatocellular carcinoma (HCC). This study aimed to compare OS after RFA with that after resection for HCC. This retrospective study included patients who underwent RFA or surgical resection as initial treatment for hepatitis B virus (HBV)-related HCC at a very early or early stage. A total of 761 patients (RFA, *n* = 194; resection, *n* = 567) from Seoul National University Hospital (Seoul, South Korea) and 1277 patients (RFA, *n* = 352; resection, *n* = 925) from the Korean Primary Liver Cancer Registry were included in the hospital and nationwide cohorts, respectively. Primary and secondary endpoints were OS and recurrence-free survival (RFS), respectively. Additional analysis was performed when the history of the antiviral treatment and the type of prescribed nucleos(t)ide analogue were confirmed. The rate of complications was compared between the two treatment groups in the hospital cohort. Baseline characteristics were balanced, using inverse probability of treatment weighting (IPTW). In the hospital cohort, the RFA group had a smaller mean tumor size (1.7 vs. 3.9 cm) but a higher proportion of cirrhotic patients than the resection group (85.6% vs. 63.1%) (both *p* < 0.01). During 81.0 (interquartile range, 62.3–107.1) months of follow-up, there was no difference in OS (adjusted hazard ratio (aHR) = 0.870, 95% confidence interval (CI) = 0.400–1.897, *p* = 0.73) and RFA was associated with shorter RFS (aHR = 1.562, 95% CI = 1.099–2.219, *p* = 0.01) after employing IPTW. Antiviral treatment was independently associated with longer OS (aHR = 0.444, 95% CI = 0.251–0.786, *p* = 0.01) as well as RFS (aHR = 0.544, 95% CI = 0.391–0.757, *p* < 0.01) in the hospital cohort. In the nationwide cohort, there was no difference in OS (aHR = 0.981, 95% CI = 0.661–1.456, *p* = 0.92) between the two treatment groups when adjusted for antiviral treatment, which was a negative independent risk factor for mortality (aHR = 0.655, 95% CI = 0.451–0.952, *p* = 0.03) after IPTW. Among patients treated with tenofovir (n = 96) or entecavir (n = 184) in the hospital cohort, there was no difference in either OS (aHR = 0.522, 95% CI = 0.058–4.724, *p* = 0.56) or RFS (aHR = 1.116, 95% CI = 0.738–1.688, *p* = 0.60). The overall incidence of complications was higher in the resection group (26.3%) than in the RFA group (13.9%) (*p* < 0.01). RFA may provide comparable OS to resection in the treatment of very early or early HCC with a lower rate of complications, although RFS is marginally shorter than in the resection group after adjusting for antiviral treatment. Regardless of the type of NA, antiviral treatment in patients with HBV-related HCC is strongly associated with both OS and RFS.

## 1. Introduction

Hepatocellular carcinoma (HCC) is the second leading cause of cancer-related mortality globally, with more than 850,000 patients newly diagnosed each year [1]. Since the importance of HCC surveillance has been emphasized over the decades, the number of HCC patients who are diagnosed at an early stage is increasing [2]. Surgical resection has been the treatment of choice for very early or early HCC because liver transplantation is feasible in limited cases. Recently, the use of local therapy, such as radiofrequency ablation (RFA) for early HCC with curative intent, has been growing, with several studies showing that RFA has therapeutic effectiveness compared to that of surgical resection in these patients [3,4,5,6]. However, the effectiveness of these two potentially curative treatment modalities is still controversial because no large-scale randomized-controlled trial (RCT) has been performed and studies including retrospective analyses, meta-analyses, and several small-scale RCTs showed conflicting results [7,8,9,10,11,12,13,14,15,16,17,18,19,20,21,22,23,24,25,26,27]. In observational studies, there was no difference in overall survival (OS) between surgical resection and RFA, although the former tended to yield longer recurrence-free survival (RFS) [11,14]. RFA has been shown to be associated with a shorter hospital stay and fewer major complications after treatment [15,22]. In an RCT comparing surgical resection and RFA to treat HCC with a diameter of less than 4 cm and up to two nodules, there was no difference in either OS or RFS [10]. In contrast, another RCT performed in patients with HCC satisfying the Milan criteria showed that surgical resection provided better OS as well as RFS than RFA [8]. Nevertheless, the number of patients enrolled in those trials was insufficient.

Chronic hepatitis B (CHB) is the most common etiology of HCC and still has a high prevalence, especially in the Asia-Pacific region, even after the introduction of the hepatitis B virus (HBV) vaccine [28]. HBV replication is a major element of immune-mediated liver injury, which may result in liver cirrhosis or HCC [29,30]. High viral load is associated with an increased risk of HCC development or recurrence after curative treatment [31,32]. Nucleos(t)ide analogues (NAs) inhibit HBV polymerase and suppress HBV replication [33]. NAs delay disease progression in CHB patients and induce the regression of liver cirrhosis with long-term treatment [34,35,36]. Several studies consistently showed that potent NA treatment may reduce the risk of HCC development [37,38]. In addition, NA therapy was associated with prolonged RFS and OS in patients who underwent surgical resection of HBV-related HCC [39,40]. Currently, entecavir (ETV) and tenofovir are used as first-line NAs because of their high potency and high barrier to resistance [41,42,43]. Although there is robust evidence that antiviral therapy is a crucial factor to determine the prognosis of patients with CHB-related HCC, most of the studies comparing surgical resection and RFA to treat early-stage HCC have not considered the antiviral treatment-related factors [10,13,14,19,20,21,22,23,24].

In this study, we aimed to compare the survival outcomes between surgical resection and RFA for HBV-related very early or early HCC after adjusting for antiviral treatment-related factors in both a hospital cohort and a nationwide HCC cohort. In addition, further analyses were performed to confirm whether antiviral treatment is associated with favorable outcomes and whether this differs between the types of NA. In short, we started this research to find answers to the following questions: (i) Is there a difference in survival outcomes between surgical resection and RFA in patients with HBV-related early HCC? (ii) Is antiviral treatment, including the type of NA, associated with overall survival?

## 2. Materials and Methods

### 2.1. Study Objects

This study consisted of two cohorts: a hospital cohort and a nationwide cohort. In the hospital cohort, consecutive patients who were treated with surgical resection or RFA for very early (Barcelona Clinic Liver Cancer [BCLC] stage 0, single nodule ≤ 2 cm) or early (BCLC stage A, single > 2 cm or ≤ 3 nodules with all ≤ 3 cm) HBV-related HCC at Seoul National University Hospital, a referral center in South Korea, between January 2006 and September 2016 were included [44,45]. In the nationwide cohort, patients who underwent surgical resection or RFA as initial treatment of BCLC stage 0 or A HBV-related HCC in Korea between January 2013 and December 2016 were enrolled from the Korean Primary Liver Cancer Registry (KPLCR). In Korea, more than 12,000 patients are newly diagnosed with HCC every year [46]. KPLCR included approximately 15% of the cases of newly diagnosed HCC in Korea (i.e., approximately 1800 cases per year), which were randomly sampled. The two cohorts complement each other. In the hospital cohort, various clinical information can be obtained from electronic medical records, but it is prone to selection bias. The nationwide cohort has an advantage in that the sample size is large, and the data are more representative. However, information on how the patients were followed up with after the initial treatment and the exact date of recurrence was limited in the nationwide cohort. Therefore, both cohorts were used to confirm whether the results from a single hospital were reproduced in a randomly sampled cohort.

**Figure 1:**
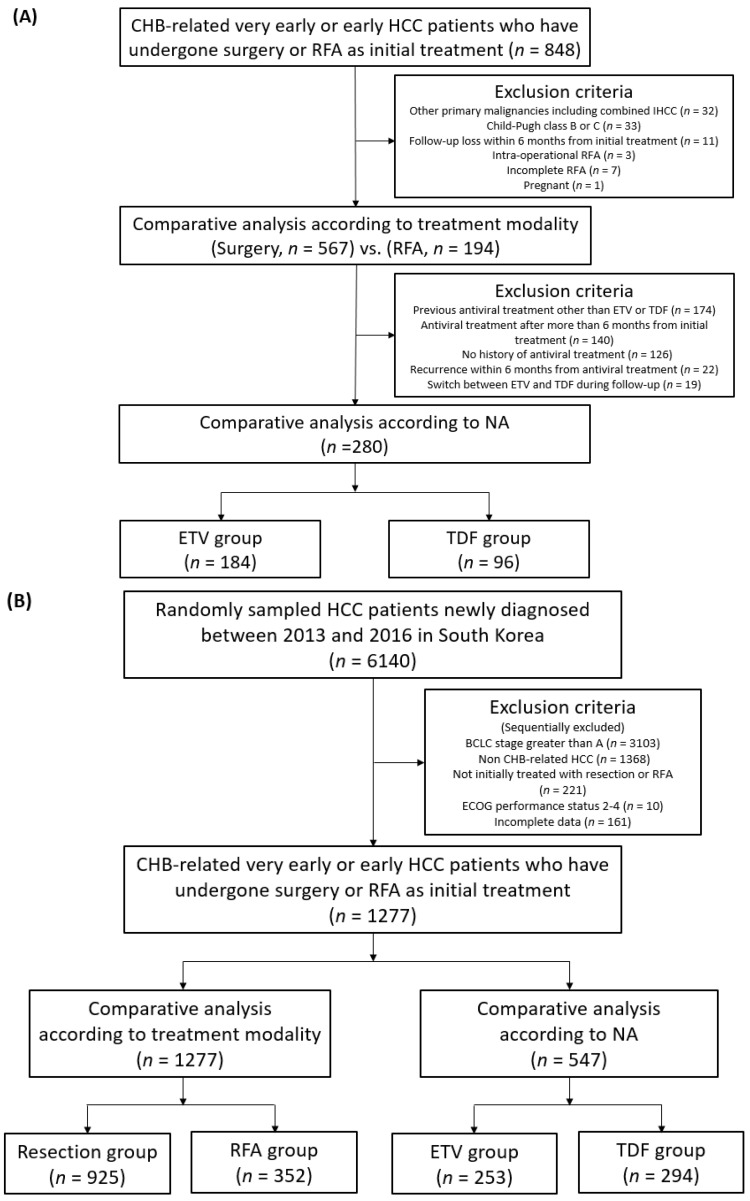
Flow diagram for (**A**) the hospital cohort and (**B**) the nationwide cohort. CHB, chronic hepatitis B; HCC, hepatocellular carcinoma; RFA, radiofrequency ablation; NA, nucleos(t)ide analogue; ETV, entecavir; TDF, tenofovir disoproxil fumarate; IHCC, intrahepatic cholangiocarcinoma; BCLC, Barcelona Clinic Liver Cancer; ECOG, European Cooperative Oncology Group.

The diagnosis of HCC was based on the guidelines from the European Association for the Study of the Liver (EASL) or the American Association for the Study of Liver Disease (AASLD) [47,48]. In the hospital cohort, the selection of the treatment modality for very early or early HCC between resection and RFA was performed by clinicians, including surgeons, radiologists, and hepatologists, through discussion. Exclusion criteria in the hospital cohort included (i) previous or current malignancies other than HCC at the time of diagnosis, including combined intrahepatic cholangiocarcinoma confirmed in a histological specimen, (ii) Child–Pugh class B or C, (iii) follow-up loss within 6 months after initial treatment, (iv) concomitant treatment with surgical resection and RFA within 2 weeks, (v) additional local treatment immediately after the initial RFA due to an incomplete procedure, and (vi) pregnancy. In the nationwide cohort, very early or early CHB-related HCC patients who were initially treated with surgical resection or RFA were included, and patients with a European Cooperative Oncology Group (ECOG) performance status of 2 or more and incomplete data were excluded. Additional analysis was performed when the history of antiviral treatment and the type of NA were confirmed in both cohorts (Figure 1).

### 2.2. Study Outcomes

The primary outcome was OS. OS was measured from the initial treatment to death from any cause. Survival data of the patients were acquired from the clinical records of the hospital, or the national database provided by the Ministry of the Interior and Safety of Korea, using the resident registration number. The secondary outcome was RFS. RFS was measured from the initial treatment to tumor recurrence or death, whichever occurred first. The data cut-off date was 31 December 2020 in the hospital cohort analysis and 31 December 2019 in the nationwide cohort analysis. After surgical resection or RFA, patients underwent scheduled imaging follow-up every 3 months for 2 years and then every 3–6 months with liver dynamic computed tomography or magnetic resonance imaging.

OS and RFS in the hospital cohort and OS in the nationwide cohort were compared according to the treatment modality and the type of NA. Subsequent analysis on a subgroup of patients with single HCC smaller than 3 cm was also performed. In addition, to verify the difference in outcomes according to antiviral therapy as well as treatment modality, patients in both cohorts were divided into four groups (patients who received both resection and antiviral treatment, RFA as well as antiviral therapy, resection alone, and RFA alone) and survival analyses were performed. The rate of complications and the length of hospital stay were compared between the two treatment groups in the hospital cohort.

### 2.3. Statistical Analysis

To compare the categorical and continuous variables, chi-squared test and independent t-test were used, respectively. OS and RFS were derived, using the Kaplan–Meier method and compared by the log-rank test. As a primary analysis, inverse probability of treatment weighting (IPTW) was used for the correction of selection bias and performed using binary logistic regression to calculate a propensity score for predicting the probability of each patient receiving a particular treatment on the basis of pretreatment baseline characteristics. The method to select relevant covariates to calculate the propensity score is described in Supplementary Methods. In addition, propensity score matching (PSM) in both cohorts was also conducted independently to determine whether the results were reproducible. For PSM, a nearest-neighbor 1:1 matching method with a caliper size of 0.1 was used. The Cox proportional hazard model was used for evaluating the independent risk factors of recurrence or survival. Variables that were clinically significant or had *p* values less than 0.1 in a univariable Cox proportional hazard model were used to perform a multivariable Cox analysis, and the hazard ratio (HR) and 95% confidence interval (CI) were calculated. Univariable and multivariable Cox analyses were performed for OS and RFS in the entire hospital cohort as well as in the subgroup of the hospital cohort with a single HCC smaller than 3 cm, and for OS in the entire nationwide cohort. OS or RFS comparison between the two treatment groups and Cox analyses were performed in an IPTW-weighted cohort, unless specified otherwise. All statistical analyses were performed using R (R 4.0.4.; R Foundation for Statistical Computing, Vienna, Austria), and *p* values were derived from two-tailed tests with a level of < 0.05 considered statistically significant.

## 3. Results

### 3.1. Baseline Characteristics

Among a total of 761 patients in the hospital cohort, 567 (74.5%) patients were treated with surgical resection (the resection group) and 194 (25.5%) patients were treated with RFA (the RFA group). Baseline characteristics according to the initial treatment modality are summarized in Table 1. The two groups differed in many baseline characteristics, including age, Child–Pugh score, BCLC stage, tumor size, cirrhosis, HBV e antigen (HBeAg) positivity, platelets, total bilirubin, albumin, and prothrombin time. The RFA group had smaller mean tumor size (1.7 vs. 3.9 cm, *p* < 0.01) and thus, a higher proportion of patients with BCLC stage 0 (58.8% vs. 16.0%, *p* < 0.01) than the resection group. The proportion of cirrhotic patients was higher in the RFA group (85.6% vs. 63.1%, *p* < 0.01), and there was no difference in the fraction of patients who were treated with antivirals, including ETV or tenofovir disoproxil fumarate (TDF) (66.0% vs. 67.2%, *p* = 0.82). Although the balance between the two groups was improved after applying IPTW, the mean tumor size (2.3 vs. 3.4 cm, *p* < 0.01, standardized mean difference (SMD) = 0.57) and the median alpha-fetoprotein (AFP) (9.9 vs. 17.6 ng/mL, *p* = 0.01, SMD = 0.17) were still smaller in the RFA group than in the resection group (Table 1).

Among a total of 1277 patients in the nationwide cohort, 925 (72.4%) patients were treated with surgical resection and 352 (27.6%) patients with RFA. The two groups differed in many variables, including age, sex, BCLC stage, tumor size, platelets, total bilirubin, albumin, prothrombin time, and tumor markers (Table S1). Similar to the hospital cohort, the RFA group had a smaller mean tumor size (1.8 vs. 3.7 cm, *p* < 0.01), which was not balanced, even after using IPTW (1.8 vs. 3.8 cm, *p* < 0.01, SMD = 0.99) (Table S1).

### 3.2. Overall and Recurrence-Free Survivals in the Entire Hospital Cohort

In the entire hospital cohort, during 81.0 (interquartile range (IQR), 62.3–107.1) months of follow-up, median OS was not reached in either group and median RFS was 71.7 months in the resection group and 40.7 months in the RFA group. Before employing IPTW, there was no difference in OS (RFA vs. resection: HR = 0.738, 95% CI = 0.435–1.250, *p* = 0.26), while the RFA group was associated with shorter RFS than the resection group (HR = 1.268, 95% CI = 1.024–1.571, *p* = 0.03).

**Figure 2:**
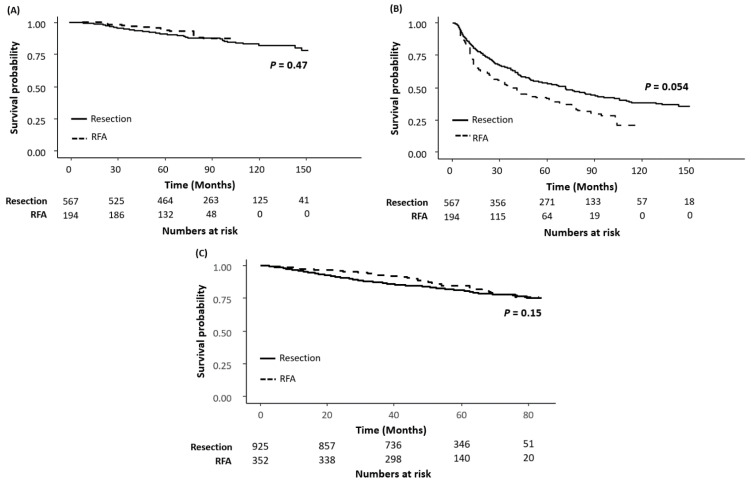
Kaplan–Meier estimates of (**A**) OS and (**B**) RFS in the hospital cohort, and (**C**) OS in the nationwide cohort after IPTW. OS or RFS was computed for all patients in the efficacy population. Patients who had not died by the date of data cut-off were censored in the case of OS estimation, and patients who had not experienced recurrence or died as of the date of data cut-off were censored in the case of RFS estimation.

After balancing baseline characteristics with IPTW, there was no difference in OS between the two groups (HR = 0.774, 95% CI = 0.368–1.630, *p* = 0.50) and RFS was longer in the resection group (HR = 1.491, 95% CI = 1.034–2.149, *p* = 0.03) (Table 2). In multivariable Cox regression analysis in the IPTW-balanced cohort, antiviral treatment including ETV or TDF was a negative independent risk factor for both OS (adjusted HR (aHR) = 0.444, 95% CI = 0.251–0.786, *p* = 0.01) and RFS (aHR = 0.544, 95% CI = 0.391–0.757, *p* < 0.01). A higher level of serum creatinine, lower level of serum albumin, larger tumor size, and the presence of varix were associated with shorter OS. A lower platelet count, the presence of cirrhosis, and multiple nodules were associated with shorter RFS. There was no difference in OS (aHR = 0.870, 95% CI = 0.400–1.897, *p* = 0.73) and RFA was associated with shorter RFS (aHR = 1.562, 95% CI = 1.099–2.219, *p* = 0.01). In the Kaplan–Meier survival analyses with the IPTW-weighted cohort, the differences in both OS (log-rank *p* = 0.47) (Figure 2A) and RFS (log-rank *p* = 0.054) (Figure 2B) curves between the two groups were not statistically significant.

After the patients in the hospital cohort were divided into four groups according to the status of initial treatment modality and antiviral therapy, OS was estimated to be shorter in patients who underwent surgery only (SR+NA−) than in those who received both surgical resection and antiviral treatment (SR+NA+) after IPTW (HR = 1.782, 95% CI = 1.077–2.949, *p* = 0.03) (Figure S1A and Table S2). The SR+NA+ group had comparable RFS to the group of patients who received both RFA and antiviral treatment (RFA + NA+) (HR = 1.337, 95% CI = 0.964–1.855, *p* = 0.08) and significantly longer RFS than the SR+NA− group (HR = 1.729, 95% CI = 1.348–2.219, *p* < 0.01) or the group of subjects who underwent RFA alone (RFA + NA−) (HR = 2.196, 95% CI = 1.525–3.162, *p* < 0.01) (Figure S1B and Table S2). When the two treatment groups in the hospital cohort were balanced using PSM, 163 pairs were matched; their baseline characteristics are summarized in Table S3. Similar to the results from the analysis of the IPTW-weighted cohort, the Kaplan–Meier analyses in the matched population showed that there was no difference in OS (log-rank *p* = 0.98) (Figure S2A) and that RFS in the resection group was significantly longer than in the RFA group (log-rank *p* = 0.02) (Figure S2B).

In the entire hospital cohort, a total of 280 patients were eligible for comparative analysis according to the type of NA. Overall, 96 (34.3%) patients and 184 (65.7%) patients were treated with TDF and ETV, respectively. Their baseline characteristics were similar, except for total bilirubin, alanine aminotransferase (ALT), prothrombin time, and HBV DNA level; all variables were well balanced after applying IPTW (Table S4). Among patients treated with TDF or ETV, there was no difference in either OS (TDF vs. ETV: aHR = 0.522, 95% CI = 0.058–4.724, *p* = 0.56) or RFS (aHR = 1.116, 95% CI = 0.738–1.688, *p* = 0.60). The Kaplan–Meier analyses showed no statistically significant differences in OS (log-rank *p* = 0.30) (Figure S3A) and RFS (log-rank *p* = 0.74) (Figure S3B) between the two groups after balancing with IPTW.

### 3.3. Survival Outcomes in the Subgroup of Single HCC Smaller Than 3 cm

In the subgroup of patients with a single HCC smaller than 3 cm, 253 (60.2%) patients were treated with surgical resection and 167 (39.8%) with RFA. The RFA group still had a smaller mean tumor size (1.6 vs. 2.1 cm) but a higher proportion of cirrhotic patients (85.6% vs. 72.7%) than the resection group (both *p* < 0.01) (Table 3). Baseline characteristics were well balanced, except for the rate of antiviral treatment, after employing IPTW (60.4% vs. 79.5%, *p* < 0.01, SMD = 0.43) (Table 3). After balancing the baseline characteristics of the two groups with IPTW, there was no difference in OS (HR = 0.735, 95% CI = 0.336–1.610, *p* = 0.44) (Table 2). Median RFS, however, was significantly shorter in the RFA group than in the resection group (40.7 vs. 90.6 months, log-rank *p* = 0.03) (Figure S4B). The RFA group showed shorter RFS in the subgroup with a single HCC smaller than 3 cm (HR = 1.628, 95% CI = 1.114–2.379, *p* = 0.01) (Table 2). In multivariable analysis, treatment modality was not an independent predictor of OS (aHR = 0.509, 95% CI = 0.213–1.215, *p* = 0.13) and RFA was associated with shorter RFS (aHR = 1.539, 95% CI = 1.057–2.242, *p* = 0.02). Antiviral treatment was an independent negative risk factor for both OS (aHR = 0.289, 95% CI = 0.141–0.585, *p* < 0.01) and RFS (aHR = 0.538, 95% CI = 0.372–0.776, *p* < 0.01).

### 3.4. Overall Survival in the Nationwide Cohort

Median OS was not reached in either treatment group during 54.0 (IQR, 43.0–67.4) months of follow-up. In the entire IPTW-balanced nationwide cohort, there was no difference in the Kaplan–Meier estimates of OS between the two groups (log-rank *p* = 0.15) (Figure S2C) as well as in the IPTW-weighted subgroup of a single HCC smaller than 3 cm (log-rank *p* = 0.39) (Figure S4C). In multivariable Cox analysis after employing IPTW, high serum albumin level (aHR = 0.511, 95% CI = 0.324–0.804, *p* < 0.01) and antiviral treatment (aHR = 0.655, 95% CI = 0.451–0.952, *p* = 0.03) were identified as independent negative risk factors (Table 2). When adjusted for age, serum albumin level, tumor size, and antiviral treatment, RFA was not associated with a higher risk of mortality, compared to surgical resection (aHR = 0.981, 95% CI = 0.661–1.456, *p* = 0.92). When patients in the nationwide cohort were divided into four groups according to the status of initial treatment and antiviral therapy, the rates of OS of the four groups were comparable (Figure S1C and Table S2). When the two treatment groups in the nationwide cohort were balanced using PSM, 347 pairs were matched; the baseline characteristics are summarized in Table S5. The Kaplan–Meier estimates of OS between the two treatment groups in the matched cohort were not statistically significant (log-rank *p* = 0.08) (Figure S2C). Although antiviral treatment was still important for survival in the nationwide cohort, there was no difference in OS rates according to the type of NA (log-rank *p* = 0.61) (Figure S3C).

### 3.5. Safety Assessment

The rate of complications after each treatment is presented in Table S6. The overall incidence of complications, including fever, biloma, bleeding, abscess, and portal vein thrombosis, was higher in the resection group (26.3%) than in the RFA group (13.9%) (*p* < 0.01). Specifically, 15 patients in the resection group received additional intervention, such as percutaneous drainage catheter insertion, pleural tapping, ascites tapping, and vascular embolization, because of the side effects after surgery. The median length of hospital stay was significantly shorter in the RFA group (3 days) than in the resection group (12 days) (*p* < 0.01).

## 4. Discussion

This retrospective cohort study analyzed 761 patients with CHB-related very early or early HCC, who were initially treated with either surgical resection or RFA. We demonstrated that there was no difference in the OS rate between the two treatment groups, and the RFA group showed shorter RFS in the entire hospital cohort as well as in the subgroup, having a single HCC smaller than 3 cm. RFA was associated with a lower rate of complications and a shorter hospital stay. Antiviral treatment, irrespective of the type of NA, was consistently verified as a strong negative risk factor for either tumor recurrence or death. These results were reproduced in the nationwide cohort, which included a total of 1277 patients with CHB-related very early or early HCC.

In both the hospital and the nationwide cohorts, RFA was performed in patients with smaller tumors. In current guidelines, RFA is the standard of care for HCC patients with BCLC stage 0 or A not suitable for surgical resection; RFA in a single tumor of 2–3 cm in size is considered an alternative treatment modality to surgical resection [47]. In patients with BCLC stage 0 HCC, RFA can be recommended as first-line therapy if the location of the tumor is favorable for the procedure. As the RFA group had a smaller tumor size than the resection group in the entire hospital cohort, which was not balanced even after employing IPTW, we performed a subgroup analysis in patients with a single HCC smaller than 3 cm. The baseline characteristics were well balanced, except for the rate of patients who received antiviral treatment after IPTW. In the multivariable Cox regression analysis adjusted for other independent risk factors including antiviral treatment, RFA was not associated with a higher risk of death and the resection group was shown to have longer RFS.

Of 567 patients in the hospital cohort who received surgical resection, 53 patients (9.3%) underwent laparoscopic surgery, and 118 patients (20.8%) received major resection (excision of three or more segments). The rate of patients receiving laparoscopic resection was relatively low because laparoscopic surgery was not common before 2010 at Seoul National University Hospital. However, the lower rate of laparoscopic surgery in this study would not have markedly affected the results of survival analyses since survival outcomes are reportedly comparable between laparoscopic resection and open hepatectomy [49,50]. In the current study, patients undergoing microwave ablation were not included, and RFA was performed by using either the switching monopolar technique with a clustered electrode or an internally cooled electrode. Further studies are required to investigate the effects of technological developments in loco-regional therapies, such as microwave ablation and multipolar RFA.

To the best of our knowledge, this is the first study comparing the effectiveness of surgical resection and RFA in the treatment of CHB-related early HCC after adjusting for antiviral treatment-related factors. Several observational studies or RCTs comparing surgical resection with RFA in patients with early HCC showed inconsistent results, and they did not take into account the effect of antiviral treatment. Now that there is sufficient evidence supporting strong associations between antiviral therapy and the recurrence and survival rates of patients with CHB-related HCC, we collected information about whether patients had received antiviral treatment, as well as the type of NA prescribed [39,40]. Antiviral treatment was consistently identified as a strong negative risk factor for recurrence or mortality throughout the study, regardless of the type of NA. The protective effect of antiviral treatment, including ETV or TDF, was confirmed in both the hospital and the nationwide cohorts. These findings are consistent with the results of previous studies showing that antiviral treatment is crucial for patients with CHB-related HCC and support the reliability of our survival analyses. The results of this study may be helpful to determine the appropriate sample sizes of future large-scale RCTs comparing the two treatment modalities because the survival rate and the number of events in CHB-related HCC patients are affected by NA treatment. Furthermore, for studies on adjuvant therapies in HCC treatment, the RFS data in this study can act as a reliable reference.

There are several limitations in this study. First, this is an observational study conducted at a single referral center, which is subject to bias and confounding factors. To overcome this problem, IPTW and multivariable adjustment were used, and the results from the hospital cohort were validated in an independent, randomly sampled nationwide HCC cohort. In both cohorts, strict inclusion and exclusion criteria were applied, and cases with incomplete data were excluded from the analyses. Second, the results from this study are limited in terms of generalizability because the study subjects were confined to patients with CHB-related HCC with a single ethnicity. However, our results are meaningful in that surgical resection and RFA were compared in a homogeneous population with identical etiology where viral factors were well controlled by NA treatment. Third, in comparing the treatment modalities, the cost-effectiveness was not considered. In a previous study comparing the cost-effectiveness of these two treatments for very early HCC and in the presence of two or three tumors measuring 3 cm or less, RFA was more cost-effective than surgical resection while surgery remained the superior modality with better survival rates at a reasonable increase in cost for a single large HCC [51]. Fourth, the baseline characteristics of the nationwide HCC cohort were not well balanced, even using IPTW, which weakens the reliability of the validation data. Although the proportions of patients who received antiviral treatment were similar between the two groups, many variables, including age, tumor size, platelets, albumin, and prothrombin time, differed in the IPTW-weighted nationwide cohort. To deal with this, we performed a subgroup analysis by limiting the tumor size, one of the most important prognostic factors, which produced similar results. To balance between the two treatment groups, IPTW rather than PSM was initially chosen to reduce the risk of false negativity and maximally utilize the available data since the number of patients in the RFA group was relatively small compared with that in the resection group. Making a perfect balance between the two groups was difficult because the number of variables (27 covariates in the hospital cohort and 19 variables in the nationwide cohort) was larger than that of other studies. However, when additional analyses in a matched cohort after PSM were performed, the baseline characteristics were better balanced, and similar results were derived as in the IPTW-weighted cohort analyses.

## 5. Conclusions

This study showed that RFA may provide comparable OS rates to surgical resection in the treatment of very early or early CHB-related HCC, although RFS is shorter after adjusting for antiviral treatment-related factors. In the RFA group, the rate of complications was lower, and the length of hospital stay was significantly shorter than in the resection group. Antiviral treatment in patients with CHB-related HCC, regardless of the type of NA, is strongly associated with both OS and RFS.

## Figures and Tables

**Table 1:** Baseline characteristics of the entire hospital cohort before and after IPTW.

Before IPTW				After IPTW			
Variables	Resection (*n* = 567)	RFA (*n* = 194)	*p*	Resection	RFA	*p*	SMD
Age, years	55.2 ± 9.5	58.3 ± 8.4	<0.01	56.1 ± 9.3	56.5 ± 9.5	0.76	0.06
Sex, male (%)	76.7	74.7	0.65	76.3	79.2	0.59	0.07
Smoking (%)	26.1	18.6	0.04	26.0	19.7	0.39	0.15
Alcohol (%)	34.4	25.8	0.03	32.9	36.7	0.62	0.08
Diabetes mellitus (%)	12.2	19.1	0.02	14.3	25.7	0.07	0.12
Hypertension (%)	27.0	26.3	0.92	27.8	32.3	0.53	0.10
Child–Pugh Score			0.01			0.55	0.06
5 (%)	90.3	82.5		88.4	90.1		
6 (%)	9.7	17.5		11.6	9.9		
BCLC stage			<0.01			0.20	0.15
0 (%)	16.0	58.8		26.9	33.9		
A (%)	84.0	41.2		73.1	66.1		
Tumor size, cm	3.9 ± 2.5	1.7 ± 0.6	<0.01	3.4 ± 2.4	2.3 ± 0.9	<0.01	0.57
Number of nodules			<0.01			0.96	0.01
1 (%)	95.8	89.7		93.7	93.6		
2 or 3 (%)	4.2	10.3		6.3	6.4		
Tumor location			0.03			0.36	0.13
Peripheral ^†^ (%)	51.0	41.8		49.0	42.6		
Central ^††^ (%)	49.0	58.2		51.0	57.4		
Perivascular tumor * (%)	28.2	21.6	0.09	26.8	18.6	0.09	0.20
Peribiliary tumor ** (%)	22.9	16.5	0.07	21.8	14.9	0.13	0.18
Presence of varix (%)	6.0	12.4	0.01	7.7	7.4	0.89	0.01
Cirrhosis (%)	63.1	85.6	<0.01	68.3	70.6	0.77	0.05
HBsAg-positive (%)	94.9	95.9	0.72	95.6	97.9	0.08	0.13
HBeAg-positive (%)	13.8	22.7	0.01	13.6	24.0	0.07	0.27
Antiviral treatment *** (%)	67.2	66.0	0.82	71.1	63.6	0.27	0.16
Platelets, ×1000/mm^3^	161.3 ± 54.0	128.8 ± 47.1	<0.01	156.0 ± 51.6	147.9 ± 43.6	0.09	0.17
Total bilirubin, mg/dL	0.9 ± 0.4	0.8 ± 0.4	<0.02	0.9 ± 0.4	0.8 ± 0.4	0.07	0.13
ALT, U/L	39.1 ± 24.1	39.3 ± 37.5	0.95	38.3 ± 23.8	40.2 ± 30.7	0.64	0.07
Albumin, g/dL	4.1 ± 0.3	4.0 ± 0.4	0.02	4.1 ± 0.4	4.1 ± 0.4	0.46	0.11
PT, INR	1.1 ± 0.1	1.1 ± 0.1	0.02	1.1 ± 0.1	1.1 ± 0.1	0.46	0.08
Serum creatinine, mg/dL	0.9 ± 0.4	0.9 ± 0.5	0.58	0.9 ± 0.3	0.9 ± 0.4	0.58	0.06
AFP, ng/mL	19.7 (4.8–317.1)	7.8 (3.7–40.8)	<0.01	17.6 (4.6–250.8)	9.9 (3.9–33.0)	0.01	0.17
PIVKA, mAU/mL	56.0 (26.0–371.5)	24.0 (17.0–35.0)	<0.01	41.0 (23.0–196.0)	27.0 (20.0–58.9)	0.06	0.03
HBV DNA, log_10_ IU/mL	2.9 ± 2.4	2.0 ± 2.5	<0.01	2.7 ± 2.4	2.5 ± 2.6	0.56	0.09

IPTW, inverse probability of treatment weighting; RFA, radiofrequency ablation; BCLC, Barcelona Clinic Liver Cancer; ALT, alanine aminotransferase; PT, prothrombin time; AFP, alpha-fetoprotein; PIVKA-II, protein induced by vitamin K absence-II; SMD, standardized mean difference. Note. Categorical variables are expressed as percentage and continuous variables as mean ± standard deviation or median (interquartile range). ^†^ Tumor located in liver segment I, II, III, VI, or VII. ^††^ Tumor located in liver segment IV, V, or VIII. * Tumor with its nearest margin ≤ 5 mm from the first- or second-degree branches of a portal or hepatic vein. ** Tumor with its nearest margin ≤ 5 mm from a common hepatic duct or main right or left hepatic duct. *** Antivirals other than entecavir or tenofovir are also included.

**Table 2:** Univariable and multivariable Cox analyses for OS and RFS in the entire hospital cohort, OS and RFS in the subgroup of the hospital cohort with a single HCC smaller than 3 cm, and OS in the entire nationwide cohort after IPTW. Hazard ratio and 95% CI were calculated, and aHR was derived after multivariable Cox analysis.

OS in the Entire Hospital Cohort				
Variables	HR	*p*	aHR	*p*
Total bilirubin	1.847 (1.045–3.265)	0.03		
Albumin	0.364 (0.162–0.822)	0.02	0.387 (0.160–0.939)	0.04
Serum creatinine	1.652 (1.499–1.819)	<0.01	1.318 (1.058–1.640)	0.01
Tumor size	1.159 (1.084–1.240)	<0.01	1.114 (1.038–1.195)	<0.01
Presence of varix	3.037 (1.633–5.649)	<0.01	2.734 (1.334–5.610)	0.01
Antiviral treatment †	0.504 (0.271–0.936)	0.03	0.444 (0.251–0.786)	0.01
RFA compared to resection	0.774 (0.368–1.630)	0.50	0.870 (0.400–1.897)	0.73
RFS in the Entire Hospital Cohort				
Variables	HR	*p*	aHR	*p*
Platelets	0.996 (0.993–0.998)	<0.01	0.996 (0.994–0.999)	0.01
Albumin	0.638 (0.431–0.945)	0.03		
Serum creatinine	1.224 (1.028–1.458)	0.02		
Tumor size	1.051 (1.006–1.098)	0.03	1.131 (1.067–1.198)	<0.01
Number of nodules ^††^	1.574 (1.142–2.169)	0.01	1.726 (1.229–2.423)	<0.01
HBeAg-positive	1.759 (1.100–2.813)	0.02	1.606 (1.039–2.480)	0.03
Cirrhosis	1.597 (1.054–2.420)	0.03	1.578 (1.020–2.440)	0.04
Presence of varix	1.491 (1.029–2.160)	0.03		
Antiviral treatment ^†^	0.555 (0.390–0.788)	<0.01	0.544 (0.391–0.757)	<0.01
RFA compared to resection	1.491 (1.034–2.149)	0.03	1.562 (1.099–2.219)	0.01
OS in the Subgroup of the Hospital Cohort with a Single HCC Smaller than 3 cm				
Variables	HR	*p*	aHR	*p*
Total bilirubin	2.296 (1.159–4.550)	0.02		
Albumin	0.310 (0.111–0.865)	0.03		
Serum creatinine	1.915 (1.676–2.189)	<0.01	2.364 (1.917–2.915)	<0.01
log_10_(HBV DNA)	1.122 (0.960–1.311)	0.15		
Presence of varix	6.806 (3.242–14.290)	<0.01	6.842 (3.030–15.447)	<0.01
Antiviral treatment ^†^	0.517 (0.239–1.121)	0.09	0.289 (0.141–0.585)	<0.01
RFA compared to resection	0.735 (0.336–1.610)	0.44	0.509 (0.213–1.215)	0.13
RFS in the Subgroup of the Hospital Cohort with a Single HCC Smaller than 3 cm				
Variables	HR	*p*	aHR	*p*
Platelets	0.995 (0.991–0.999)	0.01	0.995 (0.991–0.998)	<0.01
Total bilirubin	1.535 (1.045–2.255)	0.03		
HBeAg-positive	2.035 (1.199–3.456)	0.01	1.827 (1.142–2.290)	0.01
Tumor size	1.571 (1.009–2.446)	0.045	1.734 (1.152–2.611)	0.01
Presence of varix	1.650 (1.005–2.709)	0.048		
Antiviral treatment ^†^	0.480 (0.306–0.755)	<0.01	0.538 (0.372–0.776)	<0.01
RFA compared to resection	1.628 (1.114–2.379)	0.01	1.539 (1.057–2.242)	0.02
OS in the Entire Nationwide Cohort				
Variables	HR	*p*	aHR	*p*
Age	1.024 (1.008–1.040)	<0.01	1.022 (1.004–1.039)	0.01
Albumin	0.423 (0.301–0.594)	<0.01	0.511 (0.324–0.804)	<0.01
Child–Pugh score ^†††^	2.067 (1.414–3.021)	<0.01		
Tumor size	1.150 (1.106–1.196)	<0.01	1.148 (1.078–1.223)	<0.01
Antiviral treatment ^†^	0.620 (0.457–0.840)	<0.01	0.655 (0.451–0.952)	0.03
RFA compared to resection	0.805 (0.593–1.092)	0.16	0.981 (0.661–1.456)	0.92

RFA, radiofrequency ablation; HR, hazard ratio; aHR, adjusted hazard ratio. Note: data in parentheses are 95% confidence intervals. ^†^ Antivirals other than entecavir or tenofovir are also included. ^††^ 2–3 nodules compared to single tumor. ^†††^ Child–Pugh score 6 compared to 5.

**Table 3:** Baseline characteristics of the subgroup in the hospital cohort with a single HCC smaller than 3 cm before and after IPTW.

Before IPTW				After IPTW			
Variables	Resection (*n* = 253)	RFA (*n* = 167)	*p*	Resection	RFA	*p*	SMD
Age, years	54.4 ± 9.5	58.0 ± 8.3	<0.01	55.7 ± 9.2	55.1 ± 9.2	0.75	0.06
Sex, male (%)	74.7	73.7	0.90	74.7	79.2	0.38	0.11
Smoking (%)	28.9	19.2	0.03	28.5	25.1	0.67	0.08
Alcohol (%)	31.2	26.3	0.33	30.8	35.6	0.52	0.10
Diabetes mellitus (%)	13.0	16.8	0.36	14.4	15.6	0.79	0.03
Hypertension (%)	26.1	25.7	1.00	27.6	23.0	0.41	0.11
Child–Pugh Score			<0.01			0.84	0.02
5 (%)	92.5	82.6		88.5	89.2		
6 (%)	7.5	17.4		11.5	10.8		
BCLC stage			<0.01			0.68	0.06
0 (%)	36.0	68.3		49.1	46.2		
A (%)	64.0	31.7		50.9	53.8		
Tumor size, cm	2.1 ± 0.6	1.6 ± 0.5	<0.01	2.0 ± 0.6	2.0 ± 0.7	0.94	0.01
Tumor location			0.28			0.92	0.01
Peripheral ^†^ (%)	50.2	44.3		48.2	47.5		
Central ^††^ (%)	49.8	55.7		51.8	52.5		
Perivascular tumor * (%)	17.4	21.0	0.43	18.1	20.8	0.60	0.07
Peribiliary tumor ** (%)	13.0	16.2	0.45	13.9	16.5	0.59	0.07
Presence of varix (%)	7.9	13.2	0.11	7.4	7.7	0.90	0.01
Cirrhosis (%)	72.7	85.6	<0.01	76.6	77.5	0.88	0.02
HBsAg-positive (%)	97.6	95.8	0.44	97.7	97.6	0.94	0.01
HBeAg-positive (%)	16.6	23.4	0.11	19.5	23.4	0.54	0.10
Antiviral treatment *** (%)	77.1	64.7	0.01	79.5	60.4	<0.01	0.43
Platelets, ×1000/mm^3^	151.0 ± 47.8	126.4 ± 48.4	<0.01	145.7 ± 46.6	145.0 ± 49.4	0.90	0.02
Total bilirubin, mg/dL	0.9 ± 0.4	0.9 ± 0.4	0.10	0.9 ± 0.4	0.8 ± 0.4	0.25	0.13
ALT, U/L	37.0 ± 23.4	36.3 ± 28.0	0.78	36.1 ± 22.9	41.3 ± 28.4	0.23	0.20
Albumin, g/dL	4.1 ± 0.3	4.1 ± 0.4	0.03	4.1 ± 0.4	4.1 ± 0.4	0.36	0.11
PT, INR	1.1 ± 0.1	1.1 ± 0.1	0.71	1.1 ± 0.1	1.1 ± 0.1	0.39	0.11
Serum creatinine, mg/dL	0.9 ± 0.2	0.9 ± 0.5	0.85	0.9 ± 0.2	0.9 ± 0.4	0.42	0.08
AFP, ng/mL	16.6 (4.8–163.9)	7.1 (3.4–33.0)	<0.01	14.7 (3.9–153.2)	10.5 (3.8–89.4)	0.98	0.45
PIVKA, mAU/mL	32.0 (21.0–58.0)	23.0 (17.0–35.0)	<0.01	29.0 (4.8–317.1)	25.0 (19.0–50.1)	0.77	0.09
HBV DNA, log_10_ IU/mL	2.7 ± 2.4	1.8 ± 2.4	<0.01	2.4 ± 2.4	2.5 ± 2.6	0.84	0.03

IPTW, inverse probability of treatment weighting; RFA, radiofrequency ablation; BCLC, Barcelona Clinic Liver Cancer; ALT, alanine aminotransferase; PT, prothrombin time; AFP, alpha-fetoprotein; PIVKA-II, protein induced by vitamin K absence-II; SMD, standardized mean difference. Note. Categorical variables are expressed as percentage and continuous variables as mean ± standard deviation or median (interquartile range). ^†^ Tumor located in liver segment I, II, III, VI, or VII. ^††^ Tumor located in liver segment IV, V, or VIII. * Tumor with its nearest margin ≤ 5 mm from the first- or second-degree branches of a portal or hepatic vein. ** Tumor with its nearest margin ≤ 5 mm from a common hepatic duct or main right or left hepatic duct. *** Antivirals other than entecavir or tenofovir are also included.

## Data Availability

The data presented in this study are available in this article and Supplementary Material.

## Supplementary Materials

The following are available online at https://www.mdpi.com/article/10.3390/cancers13236009/s1, Figure S1: Kaplan–Meier estimates of (A) OS and (B) RFS in the hospital cohort, and (C) OS in the nationwide cohort according to the status of initial treatment modality and antiviral therapy after IPTW, Figure S2: Kaplan–Meier estimates of (A) OS and (B) RFS in the hospital cohort, and (C) OS in the nationwide cohort after propensity score matching, Figure S3: Kaplan–Meier estimates of (A) OS and (B) RFS in the hospital cohort, and (C) OS in the nationwide cohort according to the type of NA after IPTW, Figure S4: Kaplan–Meier estimates of (A) OS and (B) RFS in the hospital cohort, and (C) OS in the nationwide cohort for a single HCC smaller than 3 cm after IPTW, Table S1: Baseline characteristics of the entire nationwide cohort before and after IPTW, Table S2: Univariable analyses for OS as well as RFS in the hospital cohort, and OS in the nationwide cohort according to the status of initial treatment modality and antiviral therapy after IPTW, Table S3: Baseline characteristics of the entire hospital cohort before and after PSM, Table S4: Baseline characteristics according to the type of nucleos(t)ide analogues in the hospital cohort before and after IPTW, Table S5: Baseline characteristics of the entire nationwide cohort before and after PSM, Table S6: Rate of complications and hospital stay after initial treatment in the hospital cohort.
