# Supplementary material for: Comparison of Overall Survival between Surgical Resection and Radiofrequency Ablation for Hepatitis B-Related Hepatocellular Carcinoma
Source: Cancers (Basel). 2021 Nov 29;13(23):6009. doi: 10.3390/cancers13236009 (PMC8657180; doi:10.3390/cancers13236009)
Supplement: Supplementary file 1 [file cancers-13-06009-s001.zip › cancers-1476653-supplementary.pdf]

Supplementary File: Comparison of Overall Survival between Surgical Resection and Radiofrequency Ablation for Hepatitis B-Related Hepatocellular Carcinoma

Moon Haeng Hur, Jeong-Hoon Lee, Ju Yeon Kim, Ji Hoon Hong, Min Kyung Park, Hee Jin Cho, Na Ryung Choi, Jihye Kim, Minseok Albert Kim, Joon Yeul Nam, Yun Bin Lee, Eun Ju Cho, Su Jong Yu, Yoon Jun Kim, Dong Ho Lee, Jeong Min Lee, Suk Kyun Hong, Nam-Joon Yi, Kwang-Woong Lee, Kyung-Suk Suh and Jung-Hwan Yoon

Supplementary Methods: Propensity score calculation

To find variables which had statistically significant effects on overall survival (OS) or recurrence-free survival (RFS) in patients with hepatocellular carcinoma (HCC), we not only referred to the results of previous studies, but also performed Cox regression analyses in both cohorts before applying inverse probability of treatment weighting (IPTW).

In the preliminary Cox analyses before employing IPTW, male sex, larger tumor size, presence of cirrhosis, and higher serum bilirubin, aspartate transaminase (AST), alanine transaminase (ALT), prothrombin time (PT), creatinine, tumor markers, and HBV DNA were negatively associated with OS or RFS; whereas antiviral treatment, and higher platelet counts, and serum albumin level were associated with longer OS. Referring to previous studies that analyzed the variables related to survival after surgical resection or RFA in patients with HCC [1–7], propensity score was calculated using the following covariates: age, sex, diabetes mellitus status, tumor size, presence of cirrhosis, antiviral treatment, platelet counts, serum bilirubin, AST, ALT, albumin, PT, creatinine, alpha-fetoprotein (AFP), protein induced by vitamin K absence-II (PIVKA-II), and HBV DNA. We did not use different covariates according to the outcome to be analyzed (OS or RFS).

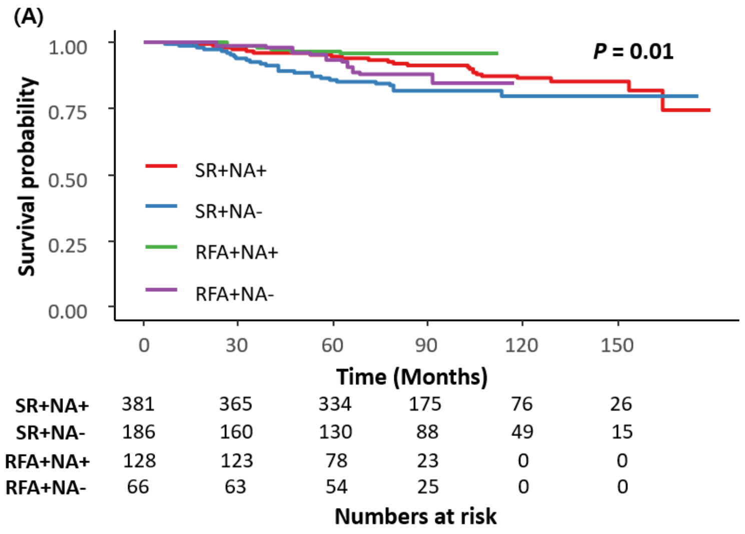

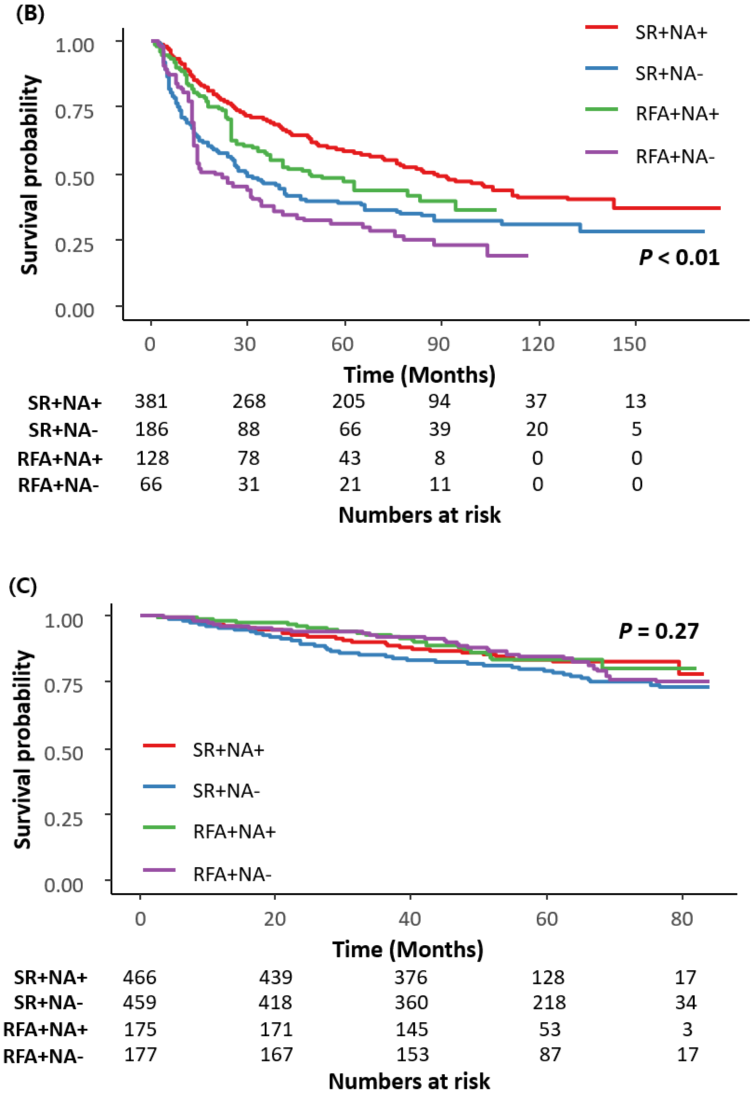

Figure S1. Kaplan-Meier estimates of (**A**) OS and (**B**) RFS in the hospital cohort, and (**C**) OS in the nationwide cohort according to the status of initial treatment modality and antiviral therapy after IPTW. SR+NA+, patients who received both surgical resection and antiviral treatment; SR+NA−, subjects who underwent surgery alone; RFA+NA+, patients who received RFA as well as antiviral therapy; RFA+NA−, a group of subjects who underwent RFA alone.

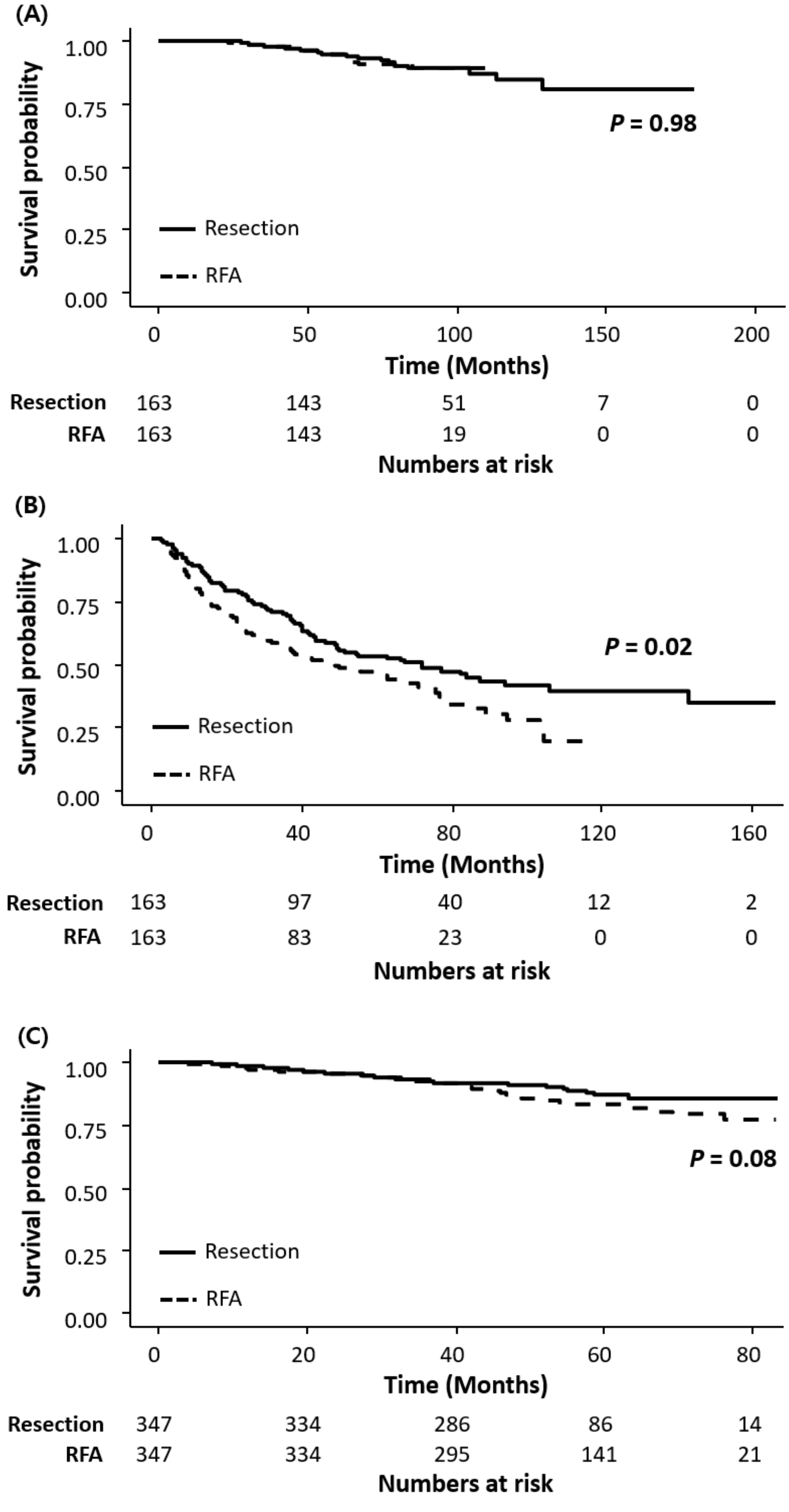

Figure S2. Kaplan-Meier estimates of (**A**) OS and (**B**) RFS in the hospital cohort, and (**C**) OS in the nationwide cohort after propensity score matching.

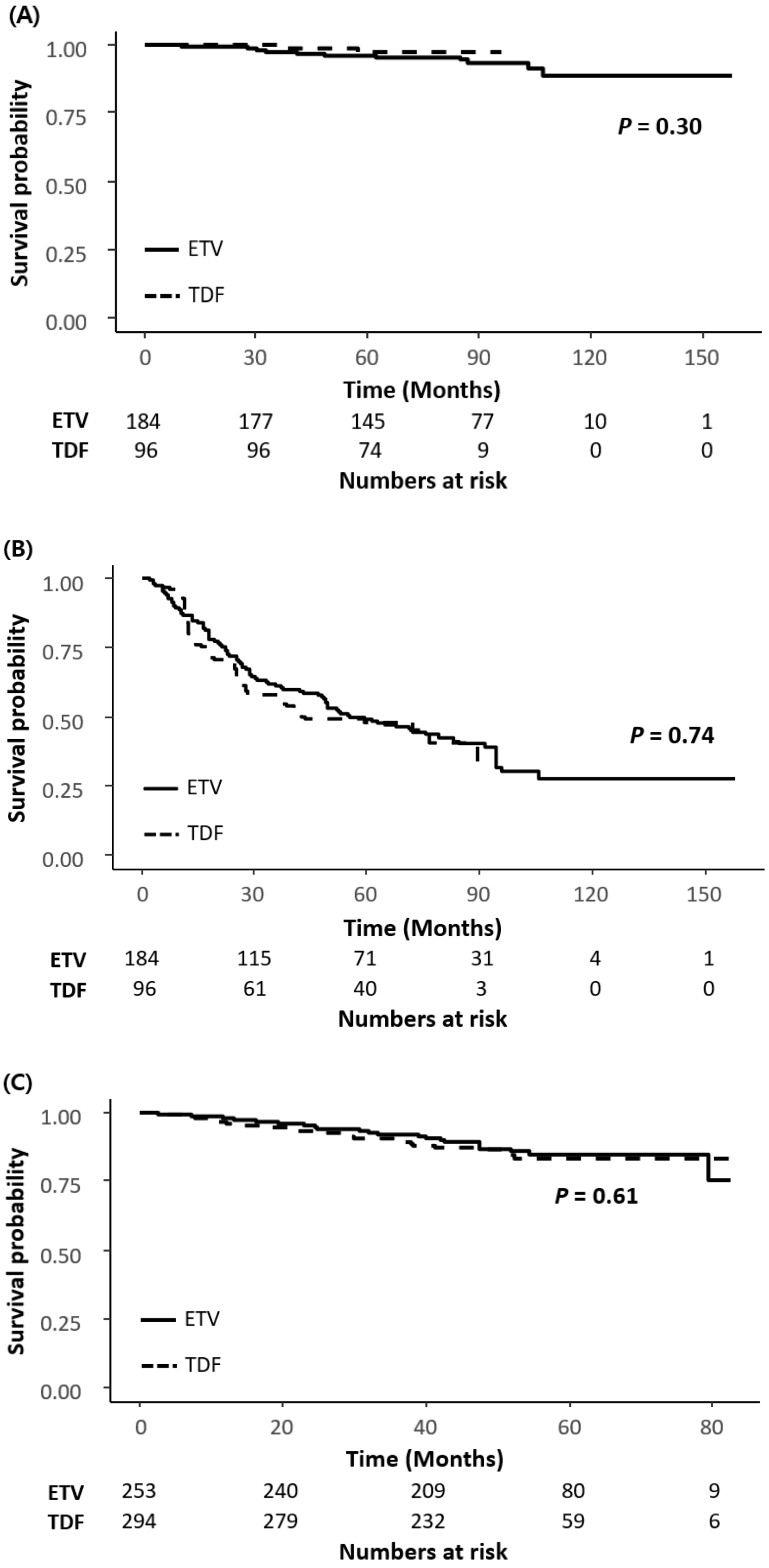

Figure S3. Kaplan-Meier estimates of (**A**) OS and (**B**) RFS in the hospital cohort, and (**C**) OS in the nationwide cohort according to the type of NA after IPTW. ETV, entecavir; TDF, tenofovir disoproxil fumarate.

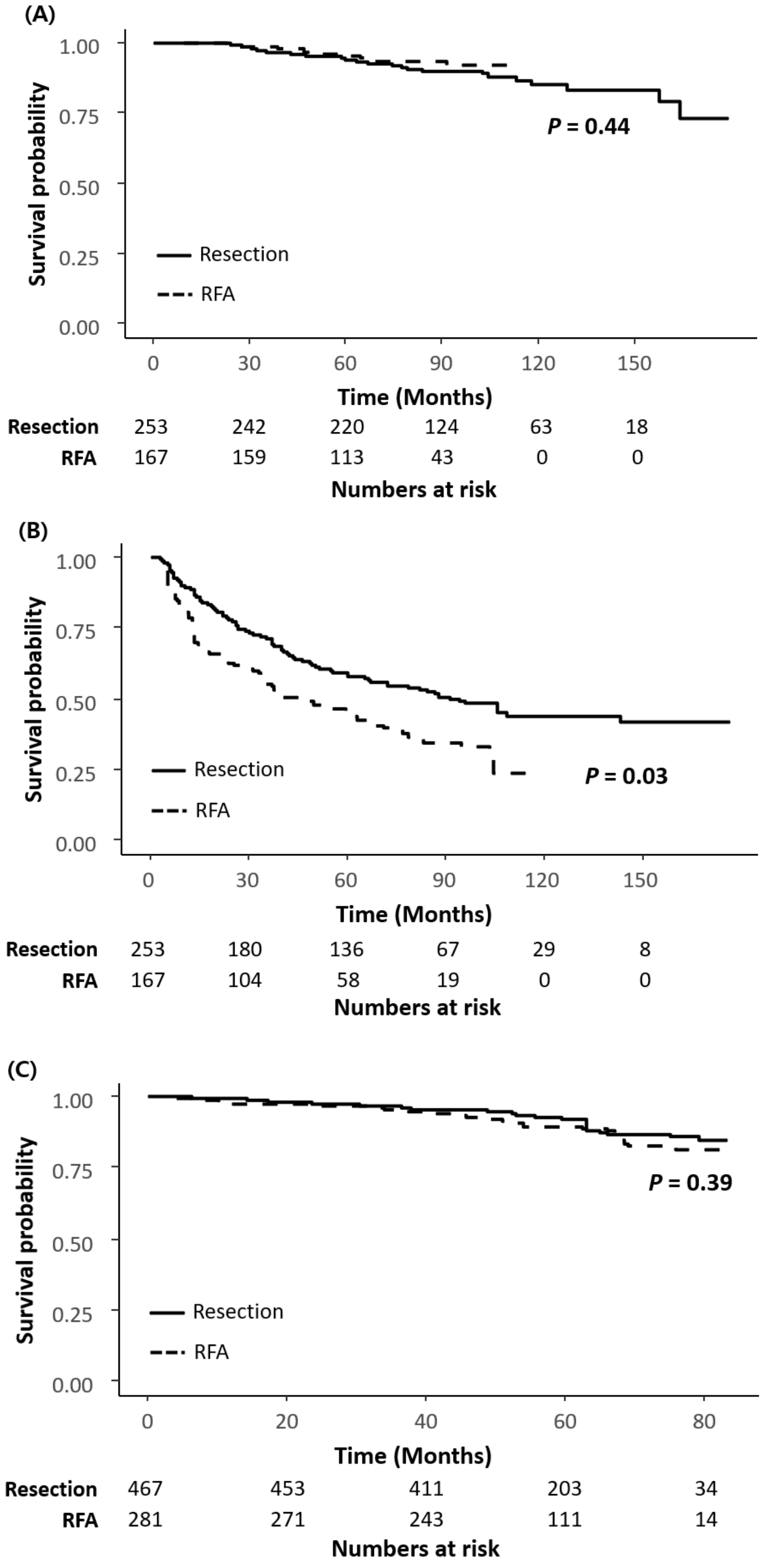

Figure S4. Kaplan-Meier estimates of (**A**) OS and (**B**) RFS in the hospital cohort, and (**C**) OS in the nationwide cohort for a single HCC smaller than 3 cm after IPTW.

Table S1. Baseline characteristics of the entire nationwide cohort before and after IPTW.

Variables	Before IPTW			After IPTW			
	Resection (*n* = 925)	RFA (*n* = 352)	*P*	Resection group	RFA group	*P*	SMD
Age, years	57.2 ± 10.1	60.9 ± 9.6	<0.01	57.3 ± 10.2	60.4 ± 9.6	<0.01	0.31
Sex, male (%)	77.8	71.6	0.02	77.7	72.2	0.06	0.13
Smoking (%)	41.4	33.0	0.01	41.0	33.9	0.04	0.15
Alcohol (%)	18.9	19.1	1.00	18.4	21.0	0.35	0.07
Diabetes mellitus (%)	22.1	24.4	0.42	21.7	25.4	0.26	0.09
Hypertension (%)	38.7	37.3	0.70	39.7	34.3	0.11	0.11
Child-Pugh Score			0.13			0.31	0.07
5 (%)	88.5	85.1		88.3	86.0		
6 (%)	11.5	14.9		11.7	14.0		
BCLC stage			<0.01			<0.01	0.95
0 (%)	22.4	64.5		22.5	65.0		
A (%)	77.6	35.5		77.5	35.0		
Tumor size, cm	3.7 ± 2.7	1.8 ± 0.8	<0.01	3.8 ± 2.8	1.8 ± 0.8	<0.01	0.99
Antiviral treatment † (%)	50.4	49.7	0.88	51.8	51.4	0.90	0.01
Platelets, ×1000/mm^3	99.7 ± 96.2	137.1 ± 55.9	<0.01	111.3 ± 97.9	135.7 ± 54.6	<0.01	0.31
Total bilirubin, mg/dL	0.8 ± 0.4	0.9 ± 0.4	0.02	0.8 ± 0.4	0.9 ± 0.4	0.06	0.13
ALT, U/L	40.5 ± 37.6	36.1 ± 42.2	0.08	40.3 ± 37.0	49.7 ± 100.6	0.55	0.13
Albumin, g/dL	4.2 ± 0.4	4.1 ± 0.4	<0.01	4.2 ± 0.4	4.1 ± 0.4	<0.01	0.24
PT, INR	1.1 ± 0.1	1.1 ± 0.1	<0.01	1.1 ± 0.1	1.1 ± 0.1	<0.01	0.28
Serum creatinine, mg/dL	0.9 ± 0.8	0.9 ± 0.3	0.09	0.9 ± 0.8	0.9 ± 0.3	0.06	0.09
AFP, ng/mL	14.5 (3.9–195.2)	6.3 (3.2–36.3)	<0.01	14.5 (3.8–210.3)	6.3 (3.2–37.7)	<0.01	0.17
PIVKA, mAU/mL	52.0 (24.0–322.5)	23.0 (16.0–34.0)	<0.01	55.0 (25.0–342.2)	22.0 (16.0–33.2)	<0.01	0.56
HBV DNA, log_{10} IU/mL	3.5 ± 1.8	3.2 ± 2.2	0.27	3.5 ± 1.8	3.2 ± 2.1	0.27	0.07

RFA, radiofrequency ablation; BCLC, Barcelona Clinic Liver Cancer; ALT, alanine aminotransferase; PT, prothrombin time; AFP, alpha-fetoprotein; PIVKA-II, protein induced by vitamin K absence-II; SMD, standardized mean difference. Note. Categorical variables are expressed as percentage and continuous variables as mean ± standard deviation or median (interquartile range). † Antivirals other than entecavir or tenofovir are also included.

Table S2. Univariable analyses for OS as well as RFS in the hospital cohort, and OS in the nationwide cohort according to the status of initial treatment modality and antiviral therapy after IPTW.

**OS in the Hospital Cohort**		
Group	HR	*P*
SR+NA+	Reference	
SR+NA-	1.782 (1.077–2.949)	0.03
RFA+NA+	0.421 (0.152–1.164)	0.10
RFA+NA-	1.400 (0.657–2.983)	0.38
RFS in the Hospital Cohort		
Group	HR	*P*
SR+NA+	Reference	
SR+NA-	1.729 (1.348–2.219)	<0.01
RFA+NA+	1.337 (0.964–1.855)	0.08
RFA+NA-	2.196 (1.525–3.162)	<0.01
OS in the Nationwide Cohort		
Group	HR	*P*
SR+NA+	Reference	
SR+NA-	1.350 (0.961–1.897)	0.08
RFA+NA+	0.958 (0.599–1.533)	0.86
RFA+NA-	1.036 (0.677–1.587)	0.87

SR+NA+, patients who received both surgical resection and antiviral treatment; SR+NA−, subjects who underwent surgery alone; RFA+NA+, patients who received RFA as well as antiviral therapy; RFA+NA−, a group of subjects who underwent RFA alone.

Table S3. Baseline characteristics of the entire hospital cohort before and after PSM.

	**Before PSM**			**After PSM**			
**Variables**	**Resection (*n* = 567)**	**RFA (*n* = 194)**	***P***	**Resection (*n* = 163)**	**RFA (*n* = 163)**	***P***	**SMD**
Age, years	55.2 ± 9.5	58.3 ± 8.4	<0.01	57.9 ± 8.0	57.8 ± 8.4	0.96	0.01
Sex, male (%)	76.7	74.7	0.65	76.1	75.5	1.00	0.01
Smoking (%)	26.1	18.6	0.04	27.6	19.0	0.09	0.20
Alcohol (%)	34.4	25.8	0.03	28.8	29.4	1.00	0.01
Diabetes mellitus (%)	12.2	19.1	0.02	19.0	18.4	1.00	0.02
Hypertension (%)	27.0	26.3	0.92	31.9	25.8	0.27	0.14
Child-Pugh Score			0.01			0.24	0.15
5 (%)	90.3	82.5		90.2	85.3		
6 (%)	9.7	17.5		9.8	14.7		
BCLC stage			<0.01			0.38	0.11
0 (%)	16.0	58.8		47.2	52.8		
A (%)	84.0	41.2		52.8	47.2		
Tumor size, cm	3.9 ± 2.5	1.7 ± 0.6	<0.01	2.0 ± 0.7	1.8 ± 0.6	0.01	0.28
Number of nodules			<0.01			0.86	0.04
1 (%)	95.8	89.7		90.2	89.0		
2 or 3 (%)	4.2	10.3		9.8	11.0		
Tumor location			0.03			0.32	0.12
Peripheral † (%)	51.0	41.8		47.9	41.7		
Central †† (%)	49.0	58.2		52.1	58.3		
Perivascular tumor * (%)	28.2	21.6	0.09	15.3	22.7	0.12	0.16
Peribiliary tumor ** (%)	22.9	16.5	0.07	13.5	17.8	0.36	0.10
Presence of varix (%)	6.0	12.4	0.01	9.2	9.8	1.00	<0.01
Cirrhosis (%)	63.1	85.6	<0.01	79.8	84.0	0.39	0.11
HBsAg-positive (%)	94.9	95.9	0.72	96.3	96.3	1.00	<0.01
HBeAg-positive (%)	13.8	22.7	0.01	14.1	23.3	0.047	0.24
Antiviral treatment *** (%)	67.2	66.0	0.82	71.8	70.6	0.90	0.03
Platelets, ×1000/mm^3	161.3 ± 54.0	128.8 ± 47.1	<0.01	138.1 ± 42.6	134.5 ± 46.4	0.46	0.08
Total bilirubin, mg/dL	0.9 ± 0.4	0.8 ± 0.4	<0.02	0.9 ± 0.4	0.8 ± 0.4	0.53	0.07

ALT, U/L	39.1 ± 24.1	39.3 ± 37.5	0.95	38.7 ± 25.5	36.3 ± 25.1	0.40	0.09
Albumin, g/dL	4.1 ± 0.3	4.0 ± 0.4	0.02	4.1 ± 0.4	4.1 ± 0.4	0.71	0.04
PT, INR	1.1 ± 0.1	1.1 ± 0.1	0.02	1.1 ± 0.1	1.1 ± 0.1	0.93	0.01
Serum creatinine, mg/dL	0.9 ± 0.4	0.9 ± 0.5	0.58	0.9 ± 0.2	0.9 ± 0.5	0.74	0.04
AFP, ng/mL	19.7 (4.8–317.1)	7.8 (3.7–40.8)	<0.01	14.7 (4.3–153.2)	8.4 (3.8–38.2)	0.02	0.22
PIVKA, mAU/mL	56.0 (26.0–371.5)	24.0 (17.0–35.0)	<0.01	29.0 (20.0–48.0)	25.0 (18.5–38.2)	0.02	0.25
HBV DNA, $\log_{10}$ IU/mL	2.9 ± 2.4	2.0 ± 2.5	<0.01	2.4 ± 2.3	2.1 ± 2.5	0.28	0.12

PSM, propensity score matching; RFA, radiofrequency ablation; BCLC, Barcelona Clinic Liver Cancer; ALT, alanine aminotransferase; PT, prothrombin time; AFP, alpha-fetoprotein; PIVKA-II, protein induced by vitamin K absence-II; SMD, standardized mean difference. Note. Categorical variables are expressed as percentage and continuous variables as mean ± standard deviation or median (interquartile range). † Tumor located in liver segment I, II, III, VI, or VII. †† Tumor located in liver segment IV, V, or VIII. * Tumor with its nearest margin ≤5 mm from the first- or second-degree branches of a portal or hepatic vein. ** Tumor with its nearest margin ≤5 mm from a common hepatic duct, main right or left hepatic duct. *** Antivirals other than entecavir or tenofovir are also included.

Table S4. Baseline characteristics according to the type of nucleos(t)ide-analogues in the hospital cohort before and after IPTW.

	**Before IPTW**			**After IPTW**			
**Variables**	**TDF (*n* = 96)**	**ETV (*n* = 184)**	***P***	**TDF**	**ETV**	***P***	**SMD**
Age, years	56.4 ± 8.1	56.2 ± 9.0	0.83	56.5 ± 7.8	55.9 ± 8.9	0.60	0.07
Sex, male (%)	80.2	78.3	0.822	79.8	78.3	0.80	0.04
Smoking (%)	24.0	22.8	0.95	21.7	25.7	0.53	0.09
Alcohol (%)	38.5	31.0	0.26	39.6	32.8	0.35	0.14
Diabetes mellitus (%)	16.7	17.9	0.92	15.9	17.1	0.82	0.03
Hypertension (%)	27.1	21.7	0.39	25.6	21.5	0.52	0.10
Child-Pugh Score			0.85			0.43	0.11
5 (%)	86.5	88.0		89.5	85.8		
6 (%)	13.5	12.0		10.5	14.2		
BCLC stage			0.27			0.09	0.24
0 (%)	24.0	31.0		21.9	32.3		
A (%)	76.0	69.0		78.1	67.7		
Tumor size, cm	2.7 ± 1.5	3.1 ± 2.2	0.12	3.1 ± 1.7	3.0 ± 2.1	0.64	0.07
Cirrhosis (%)	80.2	76.1	0.53	79.8	77.4	0.67	0.06
HBsAg-positive (%)	100.0	99.5	1.00	100	99.5	0.32	0.10
HBeAg-positive (%)	20.8	20.1	1.00	20.6	23.8	0.63	0.08
Platelets, $\times 1000/mm^3$	144.5 ± 49.5	144.5 ± 62.7	0.99	148.5 ± 47.0	139.9 ± 62.8	0.23	0.16
Total bilirubin, mg/dL	0.8 ± 0.4	0.9 ± 0.4	0.04	0.9 ± 0.4	0.9 ± 0.4	0.68	0.07
ALT, U/L	50.6 ± 31.0	36.4 ± 20.9	<0.01	41.6 ± 23.5	42.0 ± 28.3	0.94	0.01
Albumin, g/dL	4.0 ± 0.4	4.1 ± 0.4	0.06	4.1 ± 0.3	4.1 ± 0.4	0.73	0.05
PT, INR	1.1 ± 0.1	1.1 ± 0.1	0.01	1.1 ± 0.1	1.1 ± 0.1	0.97	0.01
Serum creatinine, mg/dL	0.9 ± 0.2	0.9 ± 0.5	0.55	0.9 ± 0.2	0.9 ± 0.4	0.98	<0.01
AFP, ng/mL	10.0 (4.0–97.9)	13.8 (5.2–146.0)	0.16	11.2 (4.7–105.8)	19.2 (5.2–147.3)	0.38	0.06
PIVKA, mAU/mL	33.5 (22.0–125.0)	33.0 (21.0–78.5)	0.30	31.0 (20.0–113.8)	36.0 (23.0–165.1)	0.41	0.13
HBV DNA, $\log_{10}$ IU/mL	3.6 ± 2.4	2.3 ± 2.6	<0.01	2.9 ± 2.3	2.8 ± 2.8	0.78	0.04
Initial treatment			0.21			0.70	0.06
Resection (%)	57.3	65.8		65.6	63.0		
RFA (%)	42.7	34.2		34.4	37.0		

TDF, tenofovir disoproxil fumarate; ETV, entecavir; BCLC, Barcelona Clinic Liver Cancer; ALT, alanine aminotransferase; PT, prothrombin time; AFP, alpha-fetoprotein; PIVKA-II, protein induced by vitamin K absence-II; SMD, standardized mean difference. Note. Categorical variables are expressed as percentage and continuous variables as mean ± standard deviation or median (interquartile range).

Table S5. Baseline characteristics of the entire nationwide cohort before and after PSM.

	Before PSM			After PSM			
**Variables**	**Resection (*n* = 925)**	**RFA (*n* = 352)**	***P***	**Resection (*n* = 347)**	**RFA (*n* = 347)**	***P***	**SMD**
Age, years	57.2 ± 10.1	60.9 ± 9.6	<0.01	59.1 ± 9.4	60.7 ± 9.5	0.03	0.16
Sex, male (%)	77.8	71.6	0.02	73.5	71.8	0.67	0.04
Smoking (%)	41.4	33.0	0.01	38.0	33.1	0.21	0.10
Alcohol (%)	18.9	19.1	1.00	15.0	18.7	0.22	0.10
Diabetes mellitus (%)	22.1	24.4	0.42	23.6	24.2	0.93	0.01
Hypertension (%)	38.7	37.3	0.70	44.1	36.9	0.06	0.15
Child-Pugh Score			0.13			0.36	0.08
5 (%)	88.5	85.1		88.8	86.2		
6 (%)	11.5	14.9		11.2	13.8		
BCLC stage			<0.01			<0.01	0.27
0 (%)	22.4	64.5		51.6	64.6		
A (%)	77.6	35.5		48.4	35.4		
Tumor size, cm	3.7 ± 2.7	1.8 ± 0.8	<0.01	2.1 ± 0.7	1.8 ± 0.8	<0.01	0.36
Antiviral treatment † (%)	50.4	49.7	0.88	54.5	47.6	0.08	0.14
Platelets, ×1000/mm^3	99.7 ± 96.2	137.1 ± 55.9	<0.01	129.4 ± 87.2	136.3 ± 54.2	0.21	0.10
Total bilirubin, mg/dL	0.8 ± 0.4	0.9 ± 0.4	0.02	0.8 ± 0.4	0.9 ± 0.4	0.43	0.06
ALT, U/L	40.5 ± 37.6	36.1 ± 42.2	0.08	38.8 ± 38.1	36.0 ± 42.4	0.36	0.07
Albumin, g/dL	4.2 ± 0.4	4.1 ± 0.4	<0.01	4.2 ± 0.4	4.1 ± 0.4	0.06	0.15
PT, INR	1.1 ± 0.1	1.1 ± 0.1	<0.01	1.1 ± 0.1	1.1 ± 0.1	0.13	0.12
Serum creatinine, mg/dL	0.9 ± 0.8	0.9 ± 0.3	0.09	0.9 ± 0.8	0.9 ± 0.3	0.55	0.05
AFP, ng/mL	14.5 (3.9–195.2)	6.3 (3.2–36.3)	<0.01	13.8 (4.1–121.5)	6.5 (3.4–34.9)	<0.01	0.25
PIVKA, mAU/mL	52.0 (24.0–322.5)	23.0 (16.0–34.0)	<0.01	38.0 (21.0–64.5)	28.0 (17.0–38.0)	<0.01	0.29
HBV DNA, log$_{10}$ IU/mL	3.5 ± 1.8	3.2 ± 2.2	0.27	3.2 ± 1.8	3.2 ± 2.2	0.89	0.02

PSM, propensity score matching; RFA, radiofrequency ablation; BCLC, Barcelona Clinic Liver Cancer; ALT, alanine aminotransferase; PT, prothrombin time; AFP, alpha-fetoprotein; PIVKA-II, protein induced by vitamin K absence-II; SMD, standardized mean difference. Note. Categorical variables are expressed as percentage and continuous variables as mean ± standard deviation or median (interquartile range). † Antivirals other than entecavir or tenofovir are also included.

Table S6. Rate of complications and hospital stay after initial treatment in the hospital cohort.

Variables	Resection (*n* = 567)	RFA (*n* = 194)	*P*
Overall, *n* (%)	149 (26.3)	27 (13.9)	<0.01
Fever, *n* (%)	132 (23.3)	24 (12.4)	<0.01
Biloma, *n* (%)	10 (1.8)	0 (0)	0.07
Bleeding, *n* (%)	5 (0.9)	3 (1.5)	0.43
Abscess, *n* (%)	2 (0.4)	0 (0)	1.00
PVT, *n* (%)	9 (1.6)	2 (1.0)	0.74
Complication requiring additional intervention †, *n* (%)	15 (2.8)	1 (0.5)	0.06
Hospital stay, days	12.0 (11.0–14.0)	3.0 (3.0–5.0)	<0.01

RFA, radiofrequency ablation; PVT, portal vein thrombosis. Note. The length of hospital stay is expressed as median (interquartile range) and the other variables as the number of patients (%). † Additional intervention includes percutaneous drainage catheter insertion, pleural tapping, ascites tapping, and vascular embolization.

References

1. Poon, R. T., Fan, S. T., Ng, I. O., Lo, C. M., Liu, C. L., & Wong, J. Different risk factors and prognosis for early and late intrahepatic recurrence after resection of hepatocellular carcinoma. *Cancer* **2000**, *89*(3), 500–507.
2. Lam, V. W.-T.; Ng, K. K.-C.; Chok, K. S.-H.; Cheung, T.-T.; Yuen, J.; Tung, H.; Tso, W.-K.; Fan, S.-T.; Poon, R. T., Risk factors and prognostic factors of local recurrence after radiofrequency ablation of hepatocellular carcinoma. *J. Am. Coll. Surg.* **2008,** *207*(1), 20–29.
3. Colecchia, A. Prognostic factors for hepatocellular carcinoma recurrence. *World J. Gastroenterol.* **2014**, *20*, 5935–50, doi:10.3748/wjg.v20.i20.5935.
4. Goh, B.K.P.; Teo, J.-Y.; Chan, C.-Y.; Lee, S.-Y.; Jeyaraj, P.; Cheow, P.-C.; Chow, P.; Ooi, L.L.P.J.; Chung, A.Y.F. Importance of tumor size as a prognostic factor after partial liver resection for solitary hepatocellular carcinoma: Implications on the

current AJCC staging system. *J. Surg. Oncol.* **2016**, *113*, 89–93, doi:10.1002/jso.24099.

5. N'Kontchou, G.; Mahamoudi, A.; Aout, M.; Ganne-Carrié, N.; Grando, V.; Coderc, E.; Vicaut, E.; Trinchet, J.C.; Sellier, N.; Beaugrand, M.; et al. Radiofrequency ablation of hepatocellular carcinoma: Long-term results and prognostic factors in 235 Western patients with cirrhosis. *Hepatol.* **2009**, *50*, 1475–1483, doi:10.1002/hep.23181.
6. Kim, Y.-S.; Lim, H.K.; Rhim, H.; Lee, M.W.; Choi, D.; Lee, W.J.; Paik, S.W.; Koh, K.C.; Lee, J.H.; Choi, M.S.; et al. Ten-year outcomes of percutaneous radiofrequency ablation as first-line therapy of early hepatocellular carcinoma: Analysis of prognostic factors. *J. Hepatol.* **2013**, *58*, 89–97, doi:10.1016/j.jhep.2012.09.020.
7. Huo, T.-I.; Wu, J.-C.; Lui, W.-Y.; Lee, P.-C.; Huang, Y.-H.; Chau, G.-Y.; Tsay, S.-H.; Chang, F.-Y.; Lee, S.-D. Diabetes mellitus is a recurrence-independent risk factor in patients with hepatitis B virus-related hepatocellular carcinoma undergoing resection. *Eur. J. Gastroenterol. Hepatol.* **2003**, *15*, 1203–1208, doi:10.1097/00042737-200311000-00009.
